# Clinical and immune-related factors associated with exacerbation in adults with well-controlled generalized myasthenia gravis

**DOI:** 10.3389/fimmu.2023.1177249

**Published:** 2023-05-17

**Authors:** Zhuajin Bi, Jiayang Zhan, Qing Zhang, Huajie Gao, Mengge Yang, Huizhen Ge, Mengcui Gui, Jing Lin, Bitao Bu

**Affiliations:** Department of Neurology, Tongji Hospital, Tongji Medical College, Huazhong University of Science and Technology, Wuhan, Hubei, China

**Keywords:** generalized myasthenia gravis, adult, exacerbation, predictors, anti- acetylcholine receptor antibody, lymphocyte subsets

## Abstract

**Objectives:**

To describe the clinical predictors and immune-related factors for exacerbation in adults with well-controlled generalized myasthenia gravis (GMG).

**Methods:**

We conducted a retrospective analysis of 585 adults with well-controlled GMG from our institution to explore the risk factors for exacerbation. Furthermore, propensity score matching (PSM) was used to compare the proportions of lymphocyte subsets, and the levels of immunoglobulin, complement, and anti-acetylcholine receptor antibody (AChR-ab) in the peripheral blood of 111 patients with exacerbations and 72 patients without exacerbations.

**Results:**

A total of 404 patients (69.1%) experienced at least one exacerbation, and the median (interquartile range) time to the first exacerbation was 1.5 years (0.8–3.1 years). Multivariable Cox regression analysis showed that age at onset, disease duration before enrollment, Myasthenia Gravis Foundation of America classification (MGFA) class III vs. class II, MGFA class IV-V vs. class II, AChR-ab levels, anti-muscle specific kinase antibody levels, thymus hyperplasia, prednisone plus immunosuppressants vs. prednisone treatment, and thymectomy were independent predictors for exacerbations [hazard ratio (HR) = 1.011, 1.031, 1.580, 1.429, 2.007, 2.033, 1.461, 0.798, and 0.651, respectively]. Propensity-matched analysis compared 51 patient pairs. After PSM, the peripheral blood proportions of CD3^–^CD19^+^ B cells, ratios of CD3^+^CD4^+^/CD3^+^CD8^+^ T cells, and AChR-ab levels were significantly increased, and the peripheral blood proportions of CD3^+^CD8^+^ T and CD4^+^CD25^+^CD127^low+^ regulatory T cells (Tregs) were significantly lower in patients with exacerbation than in those without exacerbation (all *p* < 0.05).

**Conclusion:**

Myasthenia gravis (MG) exacerbations were more frequent in those patients with older onset age, longer disease duration, more severe MGFA classification, positive AChR-ab, and lack of combined immunotherapy or thymectomy treatment. On the other hand, CD3^–^CD19^+^ B cells, CD3^+^CD8^+^ T cells, Tregs, and AChR-ab in peripheral blood may be involved in the course of GMG exacerbation.

## 1 Introduction

Myasthenia gravis (MG) is an autoimmune neuromuscular disorder that leads to fluctuating fatigability and weakness, which is mediated by circulating antibodies against postsynaptic membrane proteins, such as antibodies against acetylcholine receptor (AChR), muscle-specific kinase (MuSK), and low-density lipoprotein receptor (LRP4) (1). The disease course of MG is highly variable, ranging from complete stable remission to relapse, exacerbation, and even death (2). Over the last few decades, the prognosis of MG has been remarkably improved, mainly due to advances in pharmacological development, intensive care, and surgical treatment (3). Nonetheless, most MG patients, especially those with generalized myasthenia gravis (GMG), tend to experience exacerbations of symptoms over the course of the disease (4). Previous studies have primarily focused on the prognostic factors for clinical remission in patients with MG, and most studies report strong evidence that an early age at onset is predictive of a better prognosis (2, 5). More attention should be paid to the prognostic factors for exacerbation in adult-onset patients with well-controlled MG (4). Furthermore, the pathogenesis of MG is mainly related to the interaction of activated T cells with effector B cells, which drives the synthesis of pathogenic antibodies, which in turn destroy AChRs or functionally related receptors at the neuromuscular junction through a complement-mediated effect (6). However, it remains unclear whether pathogenic immune factors are involved in the process of MG exacerbations. We therefore performed a large retrospective cohort analysis of adult-onset GMG patients from our clinic to identify the clinical factors predicting MG exacerbations. We also aimed to investigate the alterations of peripheral blood lymphocyte subsets, AChR antibody (AChR-ab) and complement components in the course of exacerbation.

## 2 Materials and methods

### 2.1 Patient selection

This was a retrospective study, examining the clinical records of all GMG patients with an onset age of over 18 years who were diagnosed and treated at the Neurologic Department of Tongji Hospital from January 1989 to May 2021. The diagnosis of GMG, as previously described, was based on fluctuating generalized muscle fatigability together with a positive result on one or more of the neostigmine, repetitive nerve stimulation (RNS), and AChR-ab tests (7). All patients were well controlled and had achieved stable status, which was defined as achieving minimal manifestation status (MMS) for at least 3 months. MMS was defined as no symptoms of functional limitations of MG but some weakness on examination of the extraocular muscles (8). Patients were excluded if their age at onset was under 18 years (*n* = 958), if they had only ocular manifestations throughout follow-up (*n* = 252), if they had severe heart disease, cancer (except thymoma), or liver or kidney failure (*n* = 36), or if their medical records were incomplete (*n* = 13) or the follow-up period was less than 1 year (*n* = 28). To investigate factors that may potentially result in a first exacerbation from stable status, the patients were divided into two groups based on whether they experienced MG exacerbations throughout the study: the exacerbation group and the non-exacerbation group. MG exacerbation was defined as the reappearance or worsening of any symptoms or signs of generalized muscle weakness lasting more than 24 h (7, 9).

**Figure 1:**
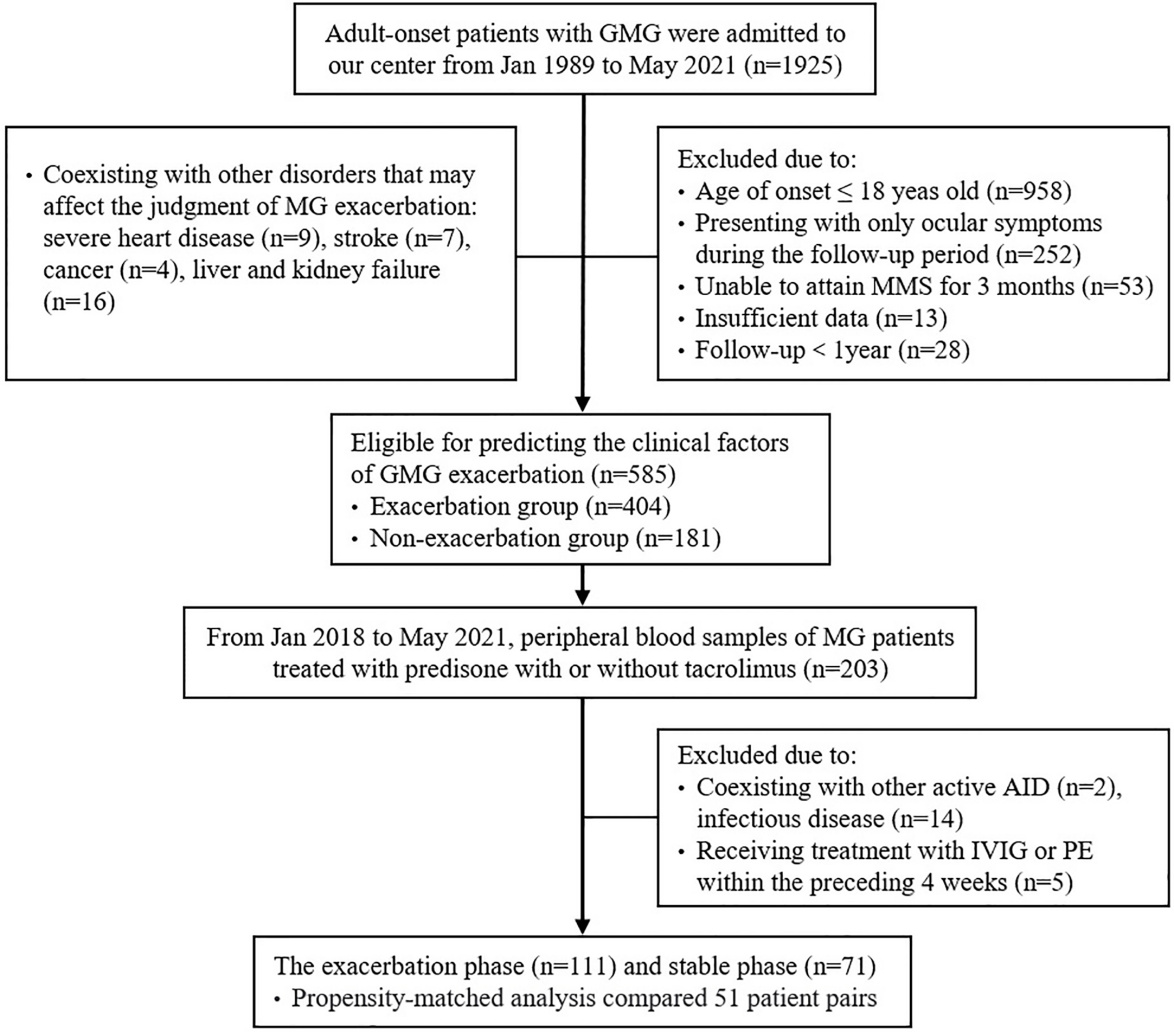
Flowchart of participants’ recruitment. AID, autoimmune disease; GMG, generalized myasthenia gravis; IVIG, intravenous immunoglobulin; MG, myasthenia gravis; MMS, minimal manifestation status; PE, plasma exchange.

In addition, to investigate alterations in peripheral blood lymphocyte subsets, immune globulin, complement components, and AChR-ab in the course of GMG exacerbation, peripheral blood samples were obtained from 203 GMG patients who were treated with prednisone, with or without tacrolimus, at Tongji Hospital from January 2018 to May 2021. During blood sample collection, patients with other active autoimmune diseases (AIDs) or infectious diseases and patients who had been treated with intravenous immunoglobulin (IVIG) or plasma exchange (PE) within the preceding 4 weeks were excluded from the analysis. Finally, peripheral blood samples were obtained from 111 patients at 3–14 days after an exacerbation (exacerbation phase) and from 72 patients who attained MMS, or better status, for more than 1 year (stable phase). To eliminate selection bias, propensity score matching (PSM) was used to pair patients in one group with another with similar baseline characteristics from the other group. Thus, the propensity-matched analysis compared 51 patient pairs. **Figure 1** outlines the selection procedure.

### 2.2 Data acquisition and evaluation

The following clinical data were collected from medical records and face-to-face interviews: age at onset, gender, disease duration, whether or not GMG symptoms presented within the first month of disease onset (limbs or bulbar predominance), the clinical classification according to Myasthenia Gravis Foundation of America (MGFA) at maximum severity (8), time from remission to the first exacerbation (if this occurred), comorbidities, results of the neostigmine or RNS test, the serum status of AChR-ab, thymus type, pharmaceutical treatments and thymectomy before the occurrence of exacerbation, and MGFA postintervention status (MGFA-PIS) at the last visit (8). The thymus status of all patients was evaluated and classified as normal (including atrophy, fatty, and cystic), hyperplastic, or thymoma based on chest computed tomography (CT) scan, magnetic resonance imaging (MRI), or histology. All chest images were read by two senior radiologists. All patients were treated with pyridostigmine and prednisone for at least 3 months before enrollment. The initial dose of prednisone was 10 mg, and the dose was increased in increments of 10 mg every 2 days, up to a dose of 1.0 mg/kg body weight, and was then gradually reduced by 5–10 mg per month after a noticeable improvement in symptoms. Immunosuppressants (IS), such as tacrolimus, azathioprine, mycophenolate mofetil, ciclosporin, and methotrexate, were given to patients who showed an unsatisfactory response, or suffered from severe adverse drug reactions to, prednisone. To better evaluate the effects of IS on MG exacerbation, IS treatment was recorded only if maintained for at least 6 months. Thymectomy was used as an alternative method based on the aspect of thymic abnormalities on chest imaging and progressive exacerbations with suboptimal response to pharmacological treatment.

The serum status of AChR-ab was estimated by enzyme-linked immunosorbent assay (ELISA) (RSR Limited, Cardiff, UK), MuSK-ab by radioimmunoassay (RIA) (RSR Limited, Cardiff, UK), Titin-ab by ELISA (DLD, Hamburg, Germany), and LRP4-ab by cell-based assays (CBA) as previously described (7, 10). AChR-ab titers > 0.50 nmol/L and MuSK-ab titers > 0.05 nmol/L were regarded as positive (RIA kit, RSR Limited). The proportions of CD3^+^CD19^–^ T cells, CD3^–^CD19^+^ T cells, CD16^+^CD56^+^ natural killer (NK) cells, CD3^+^CD4^+^ T cells, CD3^+^CD8^+^ T cells, and CD4^+^CD25^+^CD127^low+^ regulatory T cells (Tregs) were detected by flow cytometry. The serum concentrations of immune globulins (IgA, IgG, and IgM) and complement components (C3 and C4) were measured by immunoturbidimetry. The above serum immune parameters (except autoantibodies) were completed by the clinical laboratory of Tongji Hospital.

### 2.3 Statistical analysis

Numerical data are presented as mean ± standard deviation (SD) or median (interquartile range, IQR), and categorical data are presented as frequencies with absolute numbers and percentages. Differences in baseline characteristics were evaluated using the Mann–Whitney *U*-test or unpaired *t*-test when comparing unpaired continuous variables and the chi-squared or Fisher’s exact test when comparing categorical data. Exacerbation-free survival was assessed using Kaplan–Meier curves and compared using the log-rank test. A univariate Cox regression analysis was applied to identify possible factors correlated with GMG exacerbations, and variables with a *p-*value of < 0.2 were entered into the multivariate Cox regression analysis. To evaluate the immune changes that occurred during the course of MG exacerbation in patients who completed immunological tests, PSM was performed using eight variables, namely age, sex, disease duration, MGFA classification, comorbidities, thymus abnormalities, immunotherapy, and thymectomy. Patients were matched 1:1 using nearest neighbor matching with a calliper distance of 0.05. The exacerbation-related continuous variables were evaluated with receiver-operating characteristic (ROC) curves to determine appropriate cut-off values, which were defined as the maximal Youden’s index. All statistical analyses were performed with SPSS version 22.0 (SPSS Inc. Chicago, IL, USA), and a two-tailed *p* < 0.05 was considered statistically significant.

## 3 Results

### 3.1 Patient profiles

**Figure 2:**
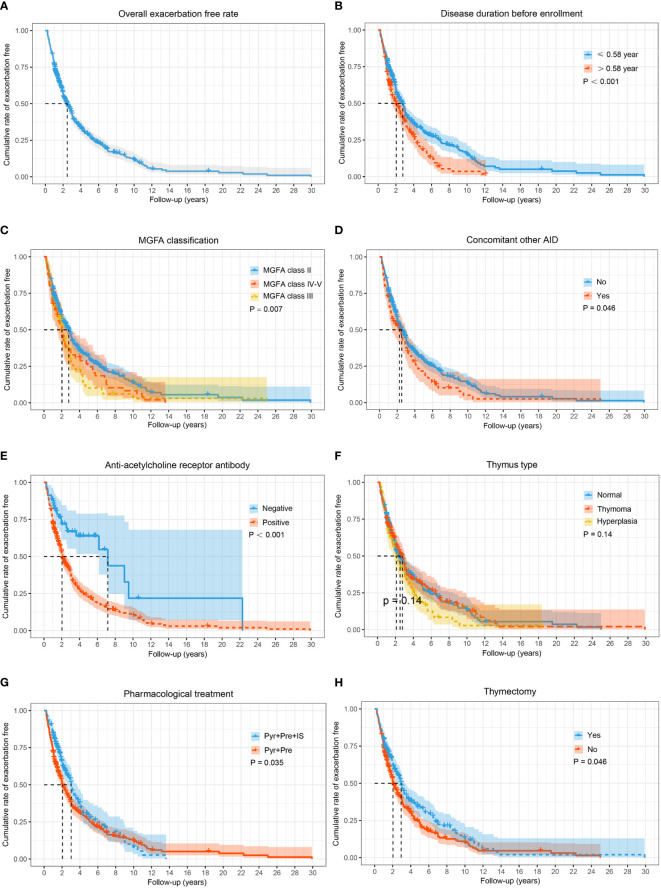
The Kaplan–Meier curve for time to exacerbate in adults with generalized MG during the entire study period. Cumulative probabilities for generalized myasthenia gravis (GMG) exacerbation-free in all patients **(A)**; cumulative probabilities for GMG exacerbation-free by stratifying patients according to disease duration before enrollment **(B)**; MGFA classification **(C)**; concomitant AID **(D)**; AChR-ab status **(E)**; thymus type **(F)**; pharmacological treatment **(G)**; and thymectomy **(H)**. A log-rank test was performed to evaluate the differences between the curves, and the two-tailed *p*-values are shown in the figure. AID, autoimmune disease; IS, immunosuppressants; MG, myasthenia gravis; MGFA, Myasthenia Gravis Foundation of America; Pre, prednisone; Pyr, pyridostigmine.

A total of 585 GMG patients (median [IQR] age at onset: 44.8 [31.0, 54.0] years; 62.6% female) were enrolled in the study (**Table 1**). At diagnosis, neostigmine testing was positive in 551 out of 585 tested cases (94.2%). Low-frequency RNS at 3 Hz was abnormal in 256 of 358 patients (71.5%). Among the patients tested for serum autoantibodies, 375 (84.5%), 14 (4.9%), 105 (36.6%) and one (1.4%) were positive for AChR-ab, MuSK-ab, Titin-ab, and LRP4-ab, respectively. Furthermore, 3 out of 282 patients (1.1%) were positive for both AChR-ab and MuSK-ab, and 101 out of 284 patients (35.6%) were positive for both AChR-ab and Titin-ab. According to the MGFA classification, there were 453 (77.4%) class II, 74 (12.6%) class III, 31 (5.3%) class IV, and 27 (4.6%) class V cases with MG. A total of 404 patients (69.1%) experienced at least one exacerbation throughout the study period, of whom 294 had one exacerbation, 81 had two exacerbations, and 29 had three or more exacerbations. The median time to the first exacerbation was 1.5 years (range 0.3–30.0 years), with 19.1% of exacerbations occurring in the 6 months after stabilization, 32.9% in the first year, and 58.9% in the first 2 years (**Figure 2A**). The 585 patients were divided into two groups: the exacerbation group (*n* = 404) and the non-exacerbation group (*n* = 181). There were significant differences between the exacerbation and non-exacerbation groups concerning disease duration before enrollment, MGFA classification, concomitant AID, AChR-ab status, thymus hyperplasia, pharmacological treatment, and thymectomy (*p* < 0.05). The best cut-off value determined in a ROC curve analysis for the disease duration before enrollment was 0.58 years (sensitivity, 34.7%; specificity, 76.2%). The Kaplan–Meier showed higher exacerbation rates and earlier time to exacerbated in those patients with longer duration (> 0.58 years), more severe MGFA classification, concomitant AID, positive AChR-ab, lack of IS or thymectomy treatment (**Figures 2B–H**) (*p* > 0.05).

**Table 1:** Baseline characteristics of 585 study participants.

Characteristics	Total (*n* = 585)	Non-exacerbation group (*n* = 181)	Exacerbation group (*n* = 404)	*p-value*
Gender Male Female	219 (37.4) 366 (62.6)	77 (42.5) 104 (57.5)	142 (35.1) 262 (64.9)	0.088
Age at onset, y^a^ EOMG (< 50 years) LOMG (≥ 50 years)	44.8 (31.0, 54.0) 377 (64.4) 208 (35.6)	43.0 (27.9, 53.5) 120 (66.3) 61 (33.7)	45.1 (32.0, 54.0) 257 (63.6) 147 (36.4)	0.222
Disease duration before enrollment, y	0.0 (0.0, 1.1)	0.0 (0.0, 0.5)	0.0 (0.0, 1.5)	0.022*
Symptom of GMG onset Limb weakness Bulbar symptoms	163 (27.9) 422 (72.1)	60 (33.1) 121 (66.9)	103 (25.5) 301 (74.5)	0.056
MGFA classification at enrollment IIA/IIB IIIA/IIIB IVA/IVB V	146/307 14/60 3/28 27	59/105 1/11 0/3 2	87/202 13/49 3/25 25	< 0.001*
Comorbidities Hypertension Diabetes Thyroid dysfunction^b^ Other AID^c^	103 (17.6) 56 (9.6) 67 (11.5) 95 (16.2)	29 (16.0) 13 (7.2) 14 (7.7) 18 (9.9)	74 (18.3) 43 (10.6) 53 (13.1) 77 (19.1)	0.501 0.188 0.059 0.006*
Neostigmine test (+)	551 (94.2)	171 (94.5)	380 (94.1)	0.843
RNS (+)	256/358	77/113	179/245	0.338
Autoantibody status AChR-ab (+) MuSK-ab (+) Titin-ab (+) LRP4-ab (+)	375/444 14/285 105/287 1/69	91/135 5/89 31/90 1/25	284/309 9/196 74/197 0/44	< 0.001* 0.710 0.611 0.181
Thymus status^d^ Normal Hyperplasia Thymoma	282 (48.2) 95 (16.2) 208 (35.6)	98 (54.1) 22 (12.2) 61 (33.7)	184 (43.5) 73 (18.1) 147 (36.4)	0.038*
Pharmacological treatment before exacerbation Pyr + Pre Pyr + Pre + IS^e^	302 (61.9) 223 (38.1)	84 (46.4) 97 (53.6)	278 (68.8) 126 (31.2)	< 0.001*
Thymectomy before exacerbation	199 (34.0)	87 (48.1)	112 (27.7)	< 0.001*
Time from onset to thymectomy, years	0.3 (0.1, 1.0)	0.3 (0.2, 1.0)	0.3 (0.1, 1.0)	0.341
MGFA-PIS at last visit CSR PR MMS Improved Unchanged Worse Exacerbation Dead	14 (2.4) 63 (10.8) 259 (44.3) 124 (21.2) 32 (5.5) 35 (6.0) 46 (7.9) 12 (2.1)	11 (6.1) 39 (21.5) 131 (72.4) 0 (0.0) 0 (0.0) 0 (0.0) 0 (0.0) 0 (0.0)	3 (0.7) 24 (5.9) 128 (31.7) 124 (30.7) 32 (7.9) 35 (8.7) 46 (11.4) 12 (3.0)	< 0.001*
Follow-up, y	2.7 (1.4, 5.1)	2.1 (1.3, 4.0)	2.9 (1.4, 5.5)	0.317

Data are given as n (%) or median (interquartile range).

Continuous data were analyzed with the Mann–Whitney U test, and categorical data were analyzed with the χ2 test or Fisher’s exact test. *: p < 0.05.

a For age at onset, the patients can be divided into early-onset (EOMG) and late-onset (LOMG) with onset before or after 50 years.

b Thyroid dysfunction included 37 cases of hyperthyroidism, 11 cases of subclinical hyperthyroidism, 12 cases of hypothyroidism, and seven cases of subclinical hypothyroidism.

^c^Concomitant autoimmune diseases included 37 cases of hyperthyroidism, 12 cases of hypothyroidism, 12 cases of rheumatics, 16 cases of rheumatoid arthritis, nine cases of systemic lupus erythematosus, five cases of Sjogren syndrome, two cases of immune-mediated necrotizing myopathies, and two cases of Guillain-Barre syndrome.

d Thymus status was evaluated by chest radiographic examination in non-thymectomized patients and histologic examination in thymectomized patients.

^e^Immunosuppressants treatment included 125 cases of tacrolimus, 67 cases of azathioprine, 18 cases of methotrexate, four cases of ciclosporin, and nine cases of mycophenolate mofetil before enrollment.

AChR-ab, anti-acetylcholine receptor antibodies; AID, autoimmune disease; CSR, complete stable remission; EOMG, early-onset MG; GMG, generalized MG; IS, immunosuppressants; LOMG, late-onset MG; MGFA, Myasthenia Gravis Foundation of America; MMS, minimal manifestation status; MuSK-ab, anti-muscle specific kinase autoantibody; NA, not available; PIS, postintervention status; Pre, prednisone; PR, pharmaceutical remission; Pyr, pyridostigmine; RNS, repetitive nerve stimulation.

### 3.2 Clinical predictors for exacerbation

Univariate Cox regression demonstrated that age at onset, disease duration before enrollment, thyroid dysfunction, concomitant AID, bulbar weakness, MGFA class III, MGFA class IV-V, AChR-ab, MuSK-ab, thymus hyperplasia, prednisone in combination with IS treatment, and thymectomy were found to be associated with the GMG exacerbation (all *p* < 0.2) (**Table 2**). Finally, multivariate Cox regression analysis indicated that age at onset (hazard ratio [HR] = 1.011, 95% confidence interval [CI] 1.004–1.018, *p* = 0.001), disease duration before enrollment (HR = 1.031, 95% CI 1.007–1.056, *p* = 0.011), MGFA class III (HR = 1.580, 95% CI 1.196–2.087, *p* = 0.001), MGFA class IV–V (HR = 1.429, 95% CI 1.059–1.928, *p* = 0.020), AChR-ab (HR = 2.007, 95% CI 1.323–3.046, *p* = 0.001), MuSK-ab (HR = 2.033, 95% CI 1.009–4.097, *p* = 0.046),and thymus hyperplasia (HR = 1.461, 95% CI 1.131–1.887, *p* = 0.004) were associated with a higher risk of GMG exacerbation, while prednisone in combination with IS treatment (HR = 0.798, 95% CI 0.643–0.989, *p* = 0.039), and thymectomy (HR = 0.651, 95% CI 0.521–0.814, *p* < 0.001) predicted the reduction of the risk of GMG exacerbation.

**Table 2:** Univariate and multivariate Cox regression analysis of predictors for GMG exacerbation.

Variable	Univariable		Multivariable	
	HR (95% CI)	*p-value*	HR (95% CI)	*p-value*
Age at onset	1.009 (1.003, 1.016)	0.006**	1.011 (1.004, 1.018)	0.001**
Female sex	1.047 (0.853, 1.285)	0.659		
Disease duration before enrollment, y	1.024 (1.001, 1.047)	0.041**	1.031 (1.007, 1.056)	0.011**
Thyroid dysfunction	1.229 (0.920, 1.642)	0.164*		
Concomitant AID	1.287 (1.003, 1.651)	0.047**		
Hypertension	1.162 (0.901, 1.499)	0.246		
Diabetes	0.974 (0.706, 1.343)	0.872		
Bulbar weakness	1.292 (1.031, 1.619)	0.026**		
MGFA classification^a^ II III IV-V	1 [Reference] 1.463 (1.110, 1.927) 1.340 (0.998, 1.797)	0.008** 0.007 0.051	1 [Reference] 1.580 (1.196, 2.087) 1.429 (1.059, 1.928)	0.001** 0.001** 0.020**
Neostigmine test (+)	0.887 (0.586, 1.342)	0.570		
RNS (+)	0.957 (0.712, 1.286)	0.770		
AChR-ab (+)^b^	2.337 (1.552, 3.520)	< 0.001**	2.007 (1.323, 3.046)	0.001** ^c^
MuSK-ab (+)^b^	1.996 (1.013, 3.934)	0.046**		
Titin-ab (+)^b^	1.193 (0.892, 1.596)	0.233		
LRP4-ab (+)^b^	NA^d^			
Thymus abnormalities Hyperplasia Thymoma	1.277 (0.999, 1.631) 0.919 (0.746, 1.133)	0.051* 0.429	1.461 (1.131, 1.887)	0.004**
Treatment^e^ Pre+IS Thymectomy	0.797 (0.644, 0.986) 0.617 (0.496, 0.768)	0.036* < 0.001**	0.798 (0.643, 0.989) 0.651 (0.521, 0.814)	0.039** < 0.001**

*p < 0.2; **p < 0.05.

a As there were a small number of patients in MGFA classes IV and V, we combined these two classes into one group and referred to them as “severe disease”.

b Analysis of patients who underwent the respective tests.

c Variables significant at univariate analysis (p < 0.2), including age at onset, disease duration before enrollment, thyroid dysfunction, concomitant AID, bulbar weakness, MGFA classification, thymus hyperplasia and treatment were examined and adjusted in multivariable analysis.

^d^Estimates are unreliable due to small number of observations.

e Treatment for patients before exacerbation (if this occurred).

AChR-ab, anti-acetylcholine receptor antibodies; AID, autoimmune disease; CI, confidence interval; HR, hazard ratio; IS, immunosuppressants; MuSK-ab, anti-muscle specific kinase autoantibody; NA, not available; Pre, prednisone; RNS, repetitive nerve stimulation.

### 3.3 Immune-related factors for GMG exacerbation

Before PSM, 182 enrolled patients were divided into two groups: 111 GMG patients in the exacerbation phase (EMG group) and 71 GMG patients in the stable phase (SMG group). There was no significant difference between the two groups in the rate of thymectomy or in the dosage and duration of prednisone and tacrolimus before blood collection (seen in **Supplement Table S1**). Thymic hyperplasia was observed more frequently in the EMG group than in the SMG group (34.2% vs. 8.5%), while tacrolimus combined with prednisone was used more frequently in the SMG group than in the EMG group (67.6% vs. 26.1%). Propensity-matched analysis compared 51 patient pairs. After matching, there was no significant difference in the proportions of CD3^+^CD19^–^ T cells, CD16^+^CD56^+^ NK cells, or CD3^+^CD4^+^ T cells, or in the levels of immune globulins (IgA, IgG, and IgM) or complement components (C3 and C4), in peripheral blood samples from patients with exacerbation and those without (**Table 3**). Notably, patients with exacerbation had a higher proportion of CD3^–^CD19^+^ B cells (14.98 ± 6.47 vs. 12.50 ± 3.94, *p* = 0.022), a higher ratio of CD3^+^CD4^+^/CD3^+^CD8^+^ T cells (2.43 ± 1.02 vs. 1.97 ± 0.97, *p* = 0.024), and a higher AChR-ab level (7.81 ± 5.27 vs. 5.37 ± 5.16, *p* = 0.020) in their peripheral blood. In addition, the proportions of CD3^+^CD8^+^ T cells (21.63 ± 6.25 vs. 26.14 ± 8.95, *p* = 0.004) and Tregs (3.14 ± 0.92 vs. 3.60 ± 0.86, *p* = 0.011) in the peripheral blood were significantly lower in the EMG group than in the SMG group.

**Table 3:** Comparison between generalized MG patients with and without exacerbation before and after PSM.

Variables	Before matching			After matching		
	EMG group (*n* = 111)	SMG group (*n* = 71)	*p-value*	EMG group (*n*=50)	SMG group (*n* = 50)	*p-value*
CD3^+^CD19^–^ T cells (%)	73.05 ± 7.45	72.84 ± 7.86	0.854	72.54 ± 7.92	73.76 ± 8.35	0.453
CD3^–^CD19^+^ B cells (%)	15.16 ± 6.42	13.82 ± 5.60	0.151	14.98 ± 6.47	12.50 ± 3.94	0.022*
CD16^+^CD56^+^ NK cells (%)	10.53 ± 6.94	12.33 ± 7.33	0.096	11.14 ± 7.81	12.76 ± 7.80	0.298
CD3^+^CD4^+^ T cells (%)	46.37 ± 8.33	42.99 ± 8.44	0.009*	47.05 ± 9.07	44.29 ± 8.64	0.119
CD3^+^CD8^+^ T cells (%)	23.16 ± 6.33	26.38 ± 8.84	0.009*	21.63 ± 6.25	26.14 ± 8.95	0.004*
CD3^+^CD4^+^/CD3^+^CD8^+^ T cells ratio	2.23 ± 0.95	1.99 ± 0.90	0.015*	2.43 ± 1.02	1.97 ± 0.97	0.024*
CD4^+^CD25^+^CD127^low–^Treg cells (%)	3.23 ± 0.97	3.61 ± 0.78	0.005*	3.14 ± 0.92	3.60 ± 0.86	0.011*
IgA (g/L)	1.99 ± 0.85	1.84 ± 0.98	0.290	2.02 ± 0.78	1.75 ± 0.89	0.106
IgG (g/L)	11.43 ± 3.06	11.20 ± 2.32	0.584	11.26 ± 2.71	11.36 ± 2.36	0.855
IgM (g/L)	1.25 ± 0.65	1.16 ± 0.60	0.350	1.37 ± 0.74	1.16 ± 0.64	0.127
C3 (g/L)	0.82 ± 0.21	0.81 ± 0.18	0.838	0.85 ± 0.26	0.82 ± 0.19	0.437
C4 (g/L)	0.20 ± 0.11	0.18 ± 0.06	0.118	0.22 ± 0.14	0.19 ± 0.06	0.177
AChR-ab (nmol/L)	8.25 ± 5.39	4.40 ± 4.70	< 0.001*	7.81 ± 5.27	5.37 ± 5.16	0.020*

Data are given as mean ± standard deviation (SD); *p < 0.05.

Analysis of immunological data was done with the two-tailed Student’s t-test.

AChR-ab, anti-acetylcholine receptor antibody; EMG, myasthenia gravis patients in exacerbation phase; MG, myasthenia gravis; NK, natural killer; PSM, propensity score matching; SMG, myasthenia gravis patients in stable phase.

## 4 Discussion

This study aims to describe the clinical characteristics of adults with GMG and to identify risk factors that can predict GMG exacerbation. Patients who had only ocular symptoms throughout the follow-up period were excluded from the analysis because such patients are clinically heterogenous (11). Therefore, the results presented in our paper are relevant only to adult-onset patients exhibiting GMG after disease onset. The results indicated that nearly 69.1% of adults with GMG experienced exacerbations, higher than the 33.7%–60.6% reported in previous literature, which may be related to higher severity and longer follow-up periods in the patients enrolled in our study (9, 12–14). Similar to other studies, most exacerbations (58.9%) occurred within the first 2 years of remission status being achieved (12, 14). The MGFA classification was identified as an independent contributor to GMG relapse, suggesting that more severe clinical symptoms tend to be associated with a higher risk of exacerbation (14). We also found, in contrast to a previous study, that gender showed noassociation with exacerbation (2). Robert et al. suggested that the presence of other AIDs was also associated with a higher risk of exacerbation (5), which indicated that MG fluctuations may be affected by the coexistence of these disorders. Notably, some medications for Graves’ disease or other comorbidities may also aggravate myasthenia symptoms (15).

Some studies have also reported that prognosis is worse among patients with thymus hyperplasia or thymoma (11, 12). Our results suggested that patients with thymic hyperplasia, but not thymoma, have a higher tendency to experience exacerbation. Further analysis revealed that thymoma may be excluded from our analysis due to its strong correlation with thymectomy (Spearman correlation coefficient = 0.686, *p* < 0.001). Cosi et al. reported that thymectomy was associated with a better prognosis only in non-thymomatous MG patients (11). On the other hand, Álvarez et al. discovered that the long-term prognosis of thymoma-associated MG patients continued to worsen even after the thymoma was removed (16). Our results suggested that successful thymectomy leads to superior long-term outcomes for patients with thymoma or thymic hyperplasia. This discrepancy may be due to the different surgical profiles of the patients, with a shorter duration from disease onset to thymectomy in our study (12). Moreover, our study has demonstrated that prednisone in combination with IS treatment leads to a reduced exacerbation rate, suggesting that additional immunotherapy contributed to maintaining long-term remission (4, 17).

It has been demonstrated that the activation and proliferation of CD4^+^ T cells is a crucial factor in the development and progression of MG diseases (6, 12). In this study, although the proportion of CD3^+^CD4^+^ T cells in the peripheral blood of patients with exacerbation was significantly higher than that of those in remission, the difference did not achieve statistical significance after PSM. Furthermore, we found that the ratio of CD3^+^/CD8^+^ T cells was significantly lower in patients with exacerbation than in those without. In addition, a high CD3^+^CD4^+^/CD3^+^CD8^+^ T cell ratio was identified as an independent risk factor for MG exacerbation. These results support the important role of equilibrium of CD3^+^CD4^+^ and CD3^+^CD8^+^ T-cell subsets in the processes of MG (18, 19). Our analysis has also revealed that Treg deficiency plays an important role in the development of MG exacerbation, which is in line with the findings of previous reports (19, 20). Tregs can be involved in the regulation of the development of MG disease mainly by secreting various cytokines and chemokines, which can promote the secretion of AChR-ab complement-binding IgG (3, 6, 19, 21). Previous studies have demonstrated that the number and activity of NK cells declines with the progression of various AIDs (22). In addition, Chien et al. discovered that the frequency of NK cells increased significantly in MG patients after plasmapheresis treatment, suggesting that NK cells might play a protective role in the development of MG (23). However, the proportion of peripheral NK cells was excluded as an independent risk factor for exacerbation in GMG exacerbation in the present study.

It is well known that humoral immunity plays a key role in the pathogenesis of MG disease by producing pathogenic autoantibodies that destroy functional AChRs at the neuromuscular junction postsynaptic membrane (23). Our data showed that the proportions of peripheral CD3^–^CD19^+^ B cells and AChR-ab levels were significantly increased in patients with MG exacerbations, suggesting that alterations in the levels of of CD3^–^CD19^+^ B cells and AChR-ab are more helpful in assessing GMG disease status (24, 25). Furthermore, among our patients, those with positive MuSK-ab were more likely to experience exacerbations. However, MuSK-ab was excluded as an independent risk factor for exacerbation after considering confounding factors, probably due to the limited number of enrolled patients in our study. There was no correlation between the clinical severity and complement levels in patients with GMG (25–27). Consistent with other studies, we found no significant association between a decrease in levels of C3 and C4 and the clinical course of MG (26, 27). In contrast, Florencia et al. suggested that C3 levels were related to the clinical severity of MG (25). Notably, because immunological investigations are performed in all GMG patients, whether or not they have experienced an exacerbation, it remains unclear whether the change in the immune biomarkers profile in the peripheral blood is a major causal event or a result of immune dysregulation that occurs during disease exacerbation.

Our study has several limitations, as follows: (1) although this was a large series, including adults with GMG demonstrating a range of distinctive characteristics throughout the clinical course, it is inevitably the case that the recruitment of subjects from a single center may result in a sample that is not entirely representative of the global population of GMG patients; (2) CT scans may have limited sensitivity to thymic hyperplasia, which leads to bias in assessing the effect of thymic abnormalities on MG exacerbation; (3) therapeutic patterns have changed with medical advances over the lengthy 30-year study period, which might have introduced bias in the evaluation of treatment outcomes; and (4) this study had a retrospective design, which may have caused unavoidable selection bias, and therefore the predictive value of immunotherapy and thymectomy needs to be further validated in large prospective controlled trials.

## 5 Conclusion

In conclusion, the majority of adults with GMG will experience at least one exacerbation throughout their life. The exacerbations tend to be more frequent in patients who were older at disease onset, in those with longer disease durations (> 0.58 years), in those with more severe disease according to the MGFA classification, in those positive for AChR-ab, and in those not treated with IS or thymectomy. On the other hand, reduced proportions of CD3^+^CD8^+^ T cells and Tregs, increased ratios of CD3^+^CD4^+^/CD3^+^CD8^+^ T cells, and high levels of AChR-ab in the peripheral blood of patients may be closely related to GMG exacerbation.

## Data availability statement

The raw data supporting the conclusions of this article will be made available by the authors, without undue reservation.

## Ethics statement

The study was approved by the Committee of Clinical Investigation at Tongji Hospital, Tongji Medical College, Huazhong University of Science and Technology, Wuhan China (NO. TJ-IRB20220748). The need for informed consent was waived due to the retrospective nature of the study. Written informed consent for participation was not required for this study in accordance with the national legislation and the institutional requirements.

## Author contributions

ZB and BB contributed to the conception and design of the study. ZB performed the statistical analysis and drafted the manuscript. ZB, JZ, QZ, HGa, MY, HGe, MG, and JL acquired the data and prepared the tables and figures. BB revised the manuscript. All authors contributed to the article and approved the submitted version.
