# Supplementary material for: Clinical and immune-related factors associated with exacerbation in adults with well-controlled generalized myasthenia gravis
Source: Front Immunol. 2023 May 17;14:1177249. doi: 10.3389/fimmu.2023.1177249 (PMC10230065; doi:10.3389/fimmu.2023.1177249)
Supplement: Supplementary file 1 [file Table_1.docx]

Supplementary Material

Clincal and immune-related factors of exacerbation in adults with well-controlled generalized myasthenia gravis

**Zhuajin Bi^1^, Jiayang Zhan^1^, Qing Zhang^1^, Huajie Gao^1^, Mengge Yang^1^, Huizhen Ge^1^, Mengcui Gui^1^, Jing Lin^1^, and Bitao Bu^1*^**

*** Correspondence:** Bitao Bu: bubitao@tjh.tjmu.edu.cn

# Supplementary Table

**Table S1**: The comparison of baseline characteristics between the EMG group and the SMG group.

| Characteristics | EMG group (n=111) | SMG group (n=71) | *P* value |
| --- | --- | --- | --- |
| Gender  Male  Female | 43 (38.7)  68 (61.3) | 29 (40.8)  42 (59.2) | 0.777 |
| Age at onset, y^a^  EOMG (< 50 y)  LOMG (≥ 50 y) | 45.5 (30.1, 53.3)  120 (66.3)  61 (33.7) | 42.8 (24.0, 56.4)  43 (60.6)  28 (39.4) | 0.758 |
| Disease duration before blood collection, y | 3.1 (1.7, 7.1) | 1.5 (1.1, 3.8) | < 0.001* |
| Symptom of GMG onset  Limb weakness  Bulbar symptoms | 35 (31.5)  76 (68.5) | 22 (31.0)  49 (69.0) | 0.938 |
| MGFA classification  II  III  IV-V | 86 (77.5)  16 (14.4)  9 (8.1) | 63 (88.7)  8 (11.3)  0 (0.0) | 0.034* |
| Neostigmine test (+) | 104 (93.7) | 69 (97.2) | 0.290 |
| RNS (+) | 54/76 | 24/34 | 0.960 |
| Autoantibody status  AChR-ab (+)  MuSK-ab (+)  Titin-ab (+) |  |  |  |
| Comorbidities  Hypertension  Diabetes  Thyroid dysfunction^b^  Other AID^c^ | 22 (19.8)  22 (19.8)  21 (18.9)  26 (23.4) | 8 (11.3)  8 (11.3)  6 (8.5)  9 (12.7) | 0.129  0.129  0.053  0.073 |
| Thymus status^d^  Normal  Hyperplasia  Thymoma | 39 (35.1)  38 (34.2)  34 (30.6) | 40 (56.3)  6 (8.5)  25 (35.2) | < 0.001* |
| Immunotherapy during blood collection  Prednisone  Prednisone +Tacrolimus | 82 (73.9)  29 (26.1) | 23 (32.4)  48 (67.6) | < 0.001* |
| Drug dosage during blood collection, mg/kg/day^e^  Prednisone  Tacrolimus | 0.17 (0.14, 0.24)  0.04 (0.04, 0.05) | 0.19 (0.12, 0.21)  0.04 (0.04, 0.04) | 0.540  0.169 |
| Duration of drug usage before blood collection, y^e^  Prednisone  Tacrolimus | 3.0 (2.2, 4.6)  2.9 (2.3, 3.5) | 3.5 (3.0, 4.9)  3.0 (2.1, 4.5) | 0.103  0.549 |
| Thymectomy | 58 (52.3) | 33 (46.5) | 0.447 |
| Time from onset to thymectomy, y | 0.9 (0.2, 4.0) | 0.4 (0.1, 1.8) | 0.073 |
| MGFA-PIS at last visit  CSR  PR  MMS  Improved  Unchanged  Worse  Exacerbation  Dead | 1 (0.9)  9 (8.1)  49 (44.1)  24 (21.6)  6 (5.4)  9 (8.1)  10 (9.0)  3 (2.7) | 3 (4.2)  19 (26.8)  49 (69.0)  0 (0.0)  0 (0.0)  0 (0.0)  0 (0.0)  0 (0.0) | < 0.001* |

Note

Data are given as n (%) or median (interquartile range).

Continuous data were analyzed with the Mann-Whitney U test, and categorical data were analyzed with the χ2 test or Fisher's exact test. ^*^ : *P* < 0.05.

^a^ As for age of onset, the patients can be divided into early-onset (EOMG) and late-onset (LOMG) with onset before or after 50 years.

^b^ Thyroid dysfunction included 18 hyperthyroidism, 2 subclinical hyperthyroidism, 6 hypothyroidism, and 1 subclinical hypothyroidism.

^c^ Concomitant autoimmune diseases included 18 hyperthyroidism, 6 hypothyroidism, 6 rheumatics, 3 rheumatoid arthritis, and 2 systemic lupus erythematosus.

^d^ Thymus status were evaluated by chest radiographic examination in non-thymectomized patients and histological examination in thymectomized patients.

^e^ Analysis of patients who received the respective treatment.

Abbreviations: AID, autoimmune disease; CSR, complete stable remission; EMG, myasthenia gravis patients in exacerbation phase; EOMG, early-onset MG; GMG, generalized MG; LOMG, late-onset MG; MGFA, Myasthenia Gravis Foundation of America; MMS, minimal manifestation status; PIS, postintervention status; PR, pharmaceutical remission; RNS, repetitive nerve stimulation; SMG, myasthenia gravis patients in stable phase.
